# Two ‘braking mechanisms’ for tin phthalocyanine molecular rotors on dipolar iron oxide surfaces

**DOI:** 10.1039/d1na00588j

**Published:** 2022-01-07

**Authors:** Shuangzan Lu, Min Huang, Guodong Huang, Qinmin Guo, Hongxing Li, Jinghao Deng, Chendong Zhang, Yinghui Yu

**Affiliations:** Department of Physics, Faculty of Physics and Electronic Sciences, Hubei University Wuhan 430062 China yhyu@hubu.edu.cn; School of Physics and Technology, Wuhan University Wuhan 430072 China cdzhang@whu.edu.cn; The State Key Laboratory of Refractories and Metallurgy, Wuhan University of Science and Technology Wuhan 430081 China; Hunan Provincial Key Laboratory of Flexible Electronic Materials Genome Engineering, Changsha University of Science and Technology Changsha 410114 China

## Abstract

Manipulation of artificial molecular rotors/motors is a key issue in the field of molecular nanomachines. Here we assemble non-planar SnPc molecules on an FeO film to form two kinds of rotors with different apparent morphologies, rotational speeds and stabilities. Both kinds of rotors can switch to each other *via* external field stimulation and the switch depends on the polarity of the applied bias voltage. Furthermore, we reveal that the molecular fragment has a great influence on the motions of molecules. Combining scanning tunneling microscopy and DFT calculations, two braking mechanisms are addressed for molecular rotors. One is the transformation of adsorption configurations under the external electric field stimulus that enables the molecular rotor to stop/restart its rotation. The other is the introduction of embedded molecular fragments that act as a brake pad and can stop the molecular rotation. We find that the rotation can be recovered by separating the molecule from the fragments. Our study suggests a good system for manipulating molecular rotors' properties in nanophysics and has important value for the design of controllable molecular machines.

## Abstract 2

Two “braking mechanisms” for molecular rotors: one is the stimuli-induced conformational change; the other is the embedded molecular fragment acting as a brake pad.

## Graphical abstract

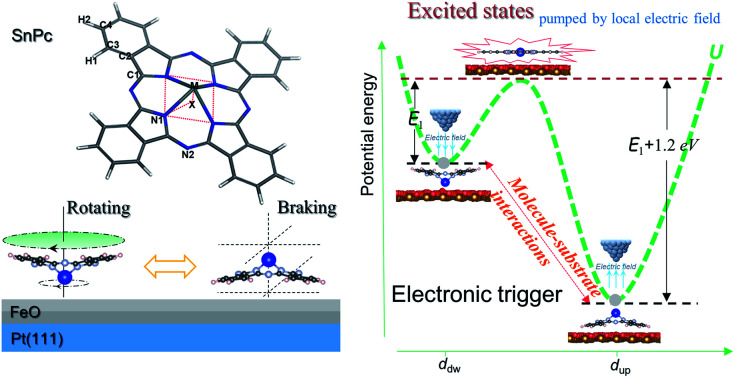

## Introduction

Fabrication and manipulation of molecular rotors and motors are crucial for artificial molecular machines,^1–5^ which will most likely be used in the fields of intelligent materials, sensors, nano-medicine and so on.^6–9^ In fact, mechanical motions at the molecular scale exist everywhere and play important roles in nature,^10,11^ for example, the kinesin protein linear motion powered by adenosine triphosphate hydrolysis,^12^ the transportation of cellular cytoplasm through vesicles and the whole bacterial locomotion driven by rotational flagella.^13^ Observations of these natural machines have attracted special interest to develop analogous artificial devices of nanoscale sizes and with controllable properties. *In vivo*, the molecular machines commonly operate at the interface,^2^ such as membrane proteins. From the perspective of bionics, surface-mounted molecular rotors are thought as one of the most promising classes of artificial devices for application in nanoscience.

Up to now, versatile molecular rotors have been successfully fabricated on solid surfaces. For example, Michl's group synthesized dipolar and nonpolar molecular rotors with an altitudinal axle.^14–18^ Gao's group constructed an array of anchored single-molecule rotors on gold surfaces.^19,20^ Zhong *et al.* exhibited surface-mounted molecular rotors with variable functional groups and rotation radii.^21^ Furthermore, Sykes' group explored the rotational mechanism of a single thioether molecule and developed a single-molecule electric motor.^22–24^ To the best of our knowledge, a small body of work explored nanoscaled molecular machines with controlled properties.^25–27^ Achieving control of molecular machines is difficult, but quite nontrivial. To realize the practical application, many problems still remain open for controlling molecular machines, such as how to adjust the rotation speed of a nanoscale molecular rotor, how to directly stop/restart the motion of a molecular motor and how to realize the propagation of rotational excitation between adjacent molecules such as a macroscopic scale gear set.^28–31^

**Fig. 1:**
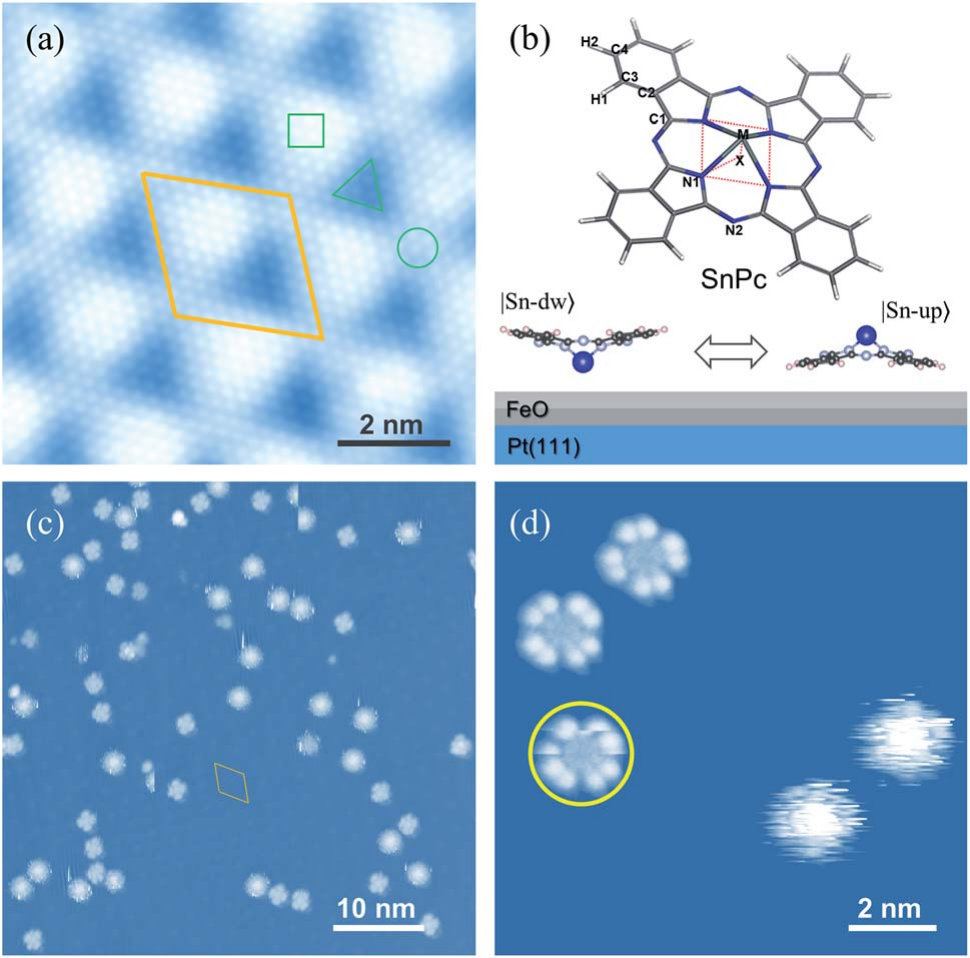
(a) Atomically-resolved STM image of nanotemplated FeO(111)/Pt(111). The Moiré unit cell is denoted by a parallelogram. Three typical sites, *i.e.* fcc, top and hcp, are labeled with △, ○ and □, respectively. (b) Chemical structure of SnPc molecules (upper panel) and schematic representation of two adsorption configurations of SnPc molecules on the FeO film (lower panel) |Sn-up〉 (Sn ion pointing to the vacuum, dipole-down) and |Sn-dw〉 (Sn ion pointing to the substrate, dipole-up). (c) Typical constant-current STM image of SnPc molecules adsorbed on the FeO surface. (d) Zoom-in STM image of low-rate (LR) and high-rate (HR) molecular rotors. The yellow circle highlights the low-rate rotor with a poor cross-shape. Here there is no nanomanipulation performed on molecules. Scale bars: 2 nm in (a) and (d) and 10 nm in (c).

In the current stage, we naturally associate the role of switches and clutches with machinery, and try to install similar functions on nanomachines. A cap-shaped tin phthalocyanine (SnPc) molecule adsorbed on the surface constitutes a single-molecule switch, with the central metal ion pointed either down to the surface or up to the vacuum with different distances (*d*) to the surface^32,33^ (Fig. 1(b)). This defines two stable structural configurations, denoted as |Sn-dw〉 and |Sn-up〉, with the adsorption energy *U*(*d*) being a double-well potential with degenerate minima at *d*_dw_ and *d*_up_. Local external stimulations are available with the tips of scanning probe microscopy. The most direct initiation of atomic motion in this switch is to exert an external stimulation on the central ion, triggering the system switch in |Sn-dw〉 and |Sn-up〉.^33^ The vertical translation of the central ion goes along with the molecular planar framework affected, *i.e.* the switching between |Sn-dw〉 and |Sn-up〉, which subtly affects the interaction between the molecular rotor and the substrate, and even influences the probability of the molecule switching between its stable states. These features inspire one to explore controlling the configuration switch and rotation speeds with local electric field applied by the metallic tip of a scanning tunneling microscope (STM). This would have great application prospects in the field of nanomechanical devices.

In this work, we systemically studied site-selective immobilization of cap-shaped dipolar SnPc molecules by STM. The FeO layer fabricated on Pt(111) is used as the nanotemplated substrate for molecular adsorption. Two types of SnPc configurations are observed and behave as molecular rotors with different rotational rates and thermal stabilities. The rotation of SnPc molecules can be stopped and restarted, and both configurations can transform into each other under the external electric-field stimulus induced by the STM tip, but with different interconversion barriers. Combining STM and density functional theory (DFT) calculations, we studied the configuration transformation in detail and suggested that the unequal interconversion barriers resulted from the total energy difference between the two configurations when adsorbing on the FeO film. Furthermore, *via* STM manipulation, we revealed that the molecular fragment has a great influence on the motions of the molecules.

## Results and discussion

Pt(111) supported FeO films are used as nanotemplates to immobilize individual SnPc molecules. The well-known lattice mismatch between Pt(111) and FeO(111) films gives rise to the Moiré patterns, as shown in Fig. 1(a). This type of self-assembled nanotemplate has a unit-cell spacing of about 2.6 nm (ref. 34) and provides an ideal geometry for the selective adsorption of SnPc molecules. The typical non-planar phthalocyanine molecule is schematically shown in the upper panel of Fig. 1(b). The centered Sn ion protrudes out of the molecular skeleton due to the large ion size. Thus, the SnPc molecules exhibit significant dipole momentum along the axis perpendicular to the molecular skeleton. In contrast to the planar metal phthalocyanines, the non-planar molecules can adsorb on a substrate surface with the central metal ions either down or up. Hence, the adsorbed configuration of non-planar SnPc could be Sn ions pointing to the substrate surface (|Sn-dw〉 with the dipole pointing to the vacuum) and to the vacuum (|Sn-up〉 with the dipole pointing to the substrate), as shown in the bottom panel of Fig. 1(b).

**Fig. 2:**
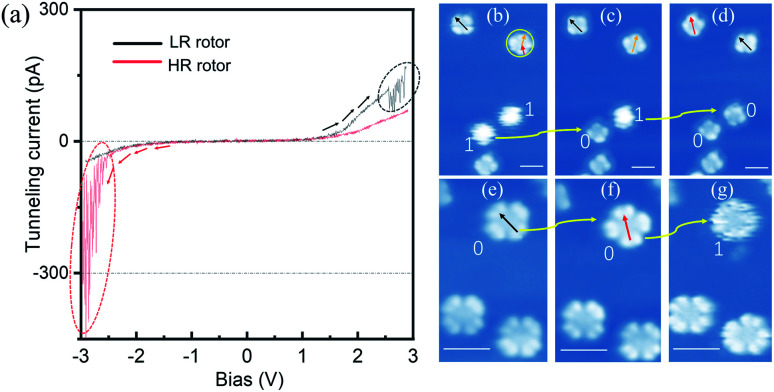
(a) Recorded *I*–*V* spectra with the STM tip triggering configuration changes. The black (red) curve is obtained on the LR (HR) rotor with the bias tuning from negative (positive) to positive (negative) regions. (b)–(d) Converting HR rotors to LR rotors one by one *via* nanomanipulation. (e)–(g) Converting LR rotors to HR rotors *via* nanomanipulation. The regions of current fluctuation are highlighted by dotted ovals. The HR (LR) rotors are labeled by numbers “1” (“0”). The colored arrows indicate three adsorption orientations of four-lobe-shaped molecules. (*V*_b_ = −1.5 V and *I*_t_ = 10 pA) Scale bars: 2 nm for (b)–(g).

Fig. 1(c) shows a typical STM image of SnPc molecules adsorbed on the FeO(111) layer. All scattered molecules are located on the fcc sites of the Moiré pattern due to the strong lateral confinements.^35^ The two adsorption configurations of SnPc exhibit distinct STM topographic features under the same tunneling conditions. As shown in a typical STM image in Fig. 1(d), two molecules located on the right panel exhibit a blurry circular shape that can be considered as the blades of a running fan blurring into a continuous circular shape.^36^ The rotational motion of the molecular configuration is faster than the scanning speed during STM imaging and thereby corresponds to the quick molecular rotation. The other molecules observed in Fig. 1(d) emerge as a four-wing structural framework with dark centers. Here each wing can itself be resolved into two separate lobes which is a characteristic STM image feature of phthalocyanines on a decoupling layer.^37^ Through detailed examination of the STM frames successively recorded, it is apparent that the orientations of four-lobe shaped molecules changed, as indicated by the colored arrows in the upper parts of Fig. 2(b)–(d). Therefore, we conclude that the four-lobe shaped molecules rotate in a much lower rate compared to the time scale of line scanning. Some four-lobed molecules marked by yellow circles show an imperfect cross-shape, since these molecules just rotate during image scanning. This observation further implies the low-rate rotation of four-lobed molecules. Hereafter, we label the four-lobe shaped molecules with dark centers as low-rate (LR) rotors and the round shaped molecules with bright centers as high-rate (HR) rotors. Obviously, both kinds of rotors rotate around the axis of the orthogonal direction due to the interactions between the adsorption site and the molecule.

To explore configuration switch of the molecular rotors, we carried out STM manipulation and current–voltage (*I*–*V*) spectral measurements. For HR rotors, the excitation energy threshold required for configuration switching was determined by positioning the STM tip above the centers of blurry circular shaped molecules and gradually increasing the bias voltage. As shown by the red curve in Fig. 2(a), the recorded *I*–*V* spectrum (where *I* and *V* are the tunneling current and the bias voltage, respectively) shows an obvious tunneling current fluctuation at about −2.30 V, which is associated with the switching process. In subsequently-recorded STM images, Fig. 2(b)–(d), the two round shaped molecules become four-lobed molecules one by one as indicated by the yellow arrows and “1” to “0”, which confirm the configuration switch from the HR to LR rotors. This transformation operation can be continuously performed with error-free conversion. Here the configuration switch should be attributed to the vertical motion of the central tin ion resulting from the electron/hole injection into molecular orbitals *via* the STM tip.^32,33^ The rotation speed of SnPc rotors could be similar to those of TiOPc rotors^35^ due to the nearly same moment of inertia of the two kinds of molecules which determine the pre-exponential frequency factor for rotation speed.^22,38–40^

Similar to the case of HR rotors, we position the STM tip above the center of LR rotors and gradually increase the tunneling voltage. The recorded *I*–*V* spectrum is shown by the black curve in Fig. 2(a). Although it also exhibits tunneling current fluctuations at the bias of ∼+2.50 V, the following recorded STM images commonly show that the LR rotor is unchanged, for example, Fig. 2(e) and (f), where the manipulated LR rotor labeled as a yellow arrow and “0” only shows an adsorption-orientation change instead of converting into the HR rotor. When positive bias voltage pulses are applied on top of the LR rotor, similar phenomena are observed and in some cases molecules are even pulsed into fragments or vanished. For only several cases of nearly a hundred attempts, the LR rotors are successfully switched to HR rotors as highlighted by “0” to “1” in Fig. 2(f) and (g). Thereby, we conclude that the transformation possibility from LR to HR rotors is very low. The reason may be that the LR rotor is inclined to convert back immediately once it is stimulated to an excited state.

Further STS measurements were carried out to clarify the influences of configuration conversion on the electronic states.

**Fig. 3:**
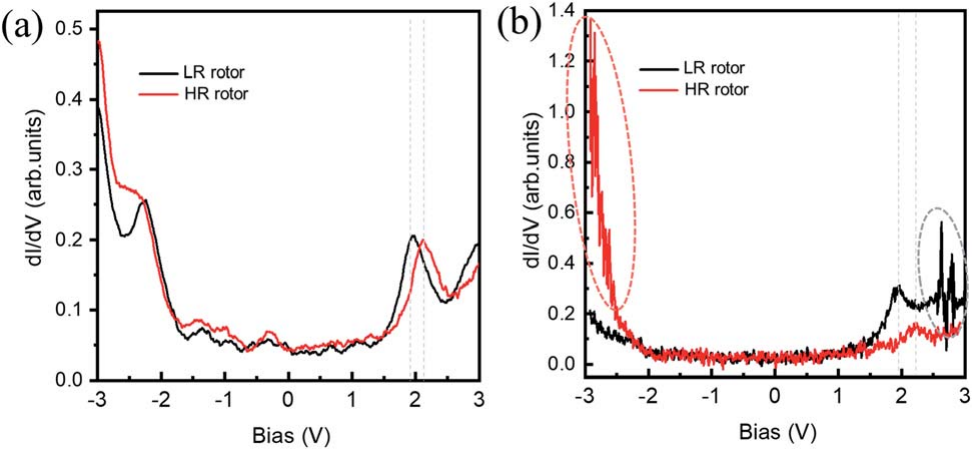
(a) d*I*/d*V* spectra obtained on the LR (black curve) and HR (red curve) rotors with a large tip-sample distance. (b) d*I*/d*V* spectra obtained on the LR (black curve) and HR (red curve) rotors with a small tip-sample distance. The regions of current fluctuation are highlighted by dotted ovals. Initial condition: *V*_b_ = −3.0 V and *I*_t_ = 50 pA for (a); *V*_b_ = −3.0 V and *I*_t_ = 400 pA for (b).

Fig. 3(a) and (b) show the d*I*/d*V* spectra recorded on the HR and LR SnPc centers with different initial tunneling currents, respectively. In Fig. 3(a), a small initial tunneling current is used to avoid triggering molecular configuration conversion. The spectra are very similar to the spectrum in the case of the SnPc monolayer on graphite,^41^ but there is a slightly larger gap value, which maybe reasonable because isolated SnPc molecules adsorb on a decoupling iron oxide layer. It is found that a distinct peak related to unoccupied molecular orbitals is located at about +2.10 V for the HR rotor, which shifts to about +1.92 V for the LR rotor. Previous studies revealed that the molecular dipole could impose a shift of the local vacuum level as well as the band alignment shifting.^42,43^ We assert that the observed offset of d*I*/d*V* spectra is related to the opposite molecular dipole directions of the two configurations. Fig. 3(b) shows the d*I*/d*V* measurements performed at a close tip-molecule separation, deliberately approaching the region of tunneling current fluctuations in order to trigger the switch. These curves record the conductance difference between HR and LR configurations and show that in the negative (positive) bias region the conductance of the HR (LR) rotors is higher (lower) than that of the LR (HR) rotors. In the negative bias region for HR rotors and the positive bias region for LR rotors, there is a significant fluctuation of the tunneling currents as highlighted by ellipses in Fig. 2(a) and 3(b). The orientations of molecular dipole moments related to the two adsorption configurations are opposite, *i.e.*, it points to the vacuum and the substrate for the |Sn-dw〉 and |Sn-up〉 molecules, respectively. As a result, the responses of the molecules to external fields depend on the polarity of the bias voltage. This should be the reason why LR to HR (HR to LR) conversion commonly occurs in the positive (negative) bias region. The two ground states of this switch labeled as “0” and “1” can be clearly distinguished in STM images due to their high on/off conductance ratio, and thus can be a single-molecule conductance switching as key elements of the molecular devices. On the FeO surface, furthermore, these single-molecule rotors can self-assemble into large-scale ordered arrays in one-monolayer coverage, which can be addressed as “read and overwritten” of high-density storage devices.

**Fig. 4:**
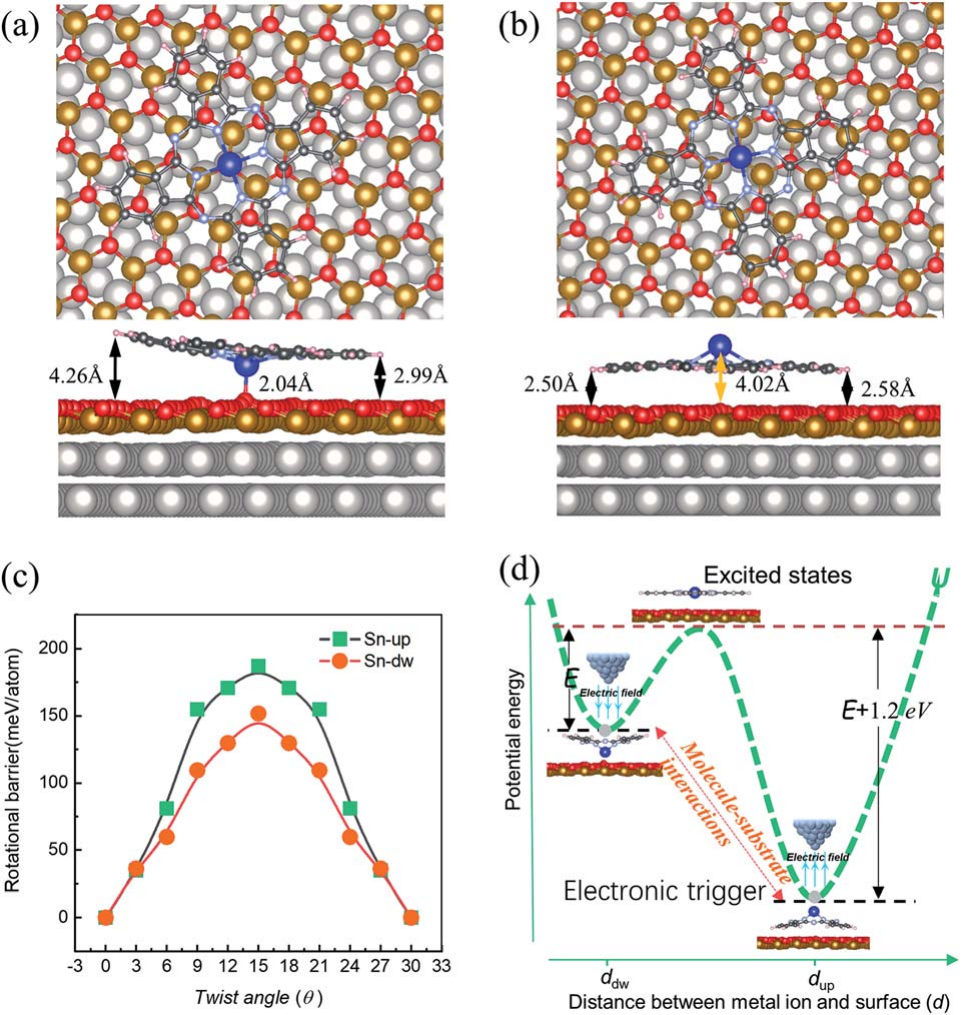
Most stable structures of SnPc molecules adsorbed at the fcc sites of FeO/Pt(111), as calculated by DFT. (a) Downward configuration and (b) upward configuration. The upper and lower panels represent the top and side views of the configurations, respectively. The large gray and gold balls represent the Pt and Fe atoms, respectively. (c) The obtained relative total energy profiles of |Sn-dw〉 and |Sn-up〉 rotors as a function of angles. (d) Schematic representation of the unequal interconversion barriers between |Sn-up〉 and |Sn-dw〉 configurations. The corresponding barriers are marked with *E* and *E* + 1.2 eV.

DFT calculations were carried out to determine the structures and energetics for both |Sn-dw〉 and |Sn-up〉 configurations. Two structures resembling the experimental conformations were found, as displayed in Fig. 4. For each configuration, there are different distances between each molecular lobe and FeO/Pt(111) surface, as shown in Fig. 4(a) and (b). The calculated average distances are associated with the different vertical positions of the tin ion and are about 3.63 Å and 2.54 Å for |Sn-dw〉 and |Sn-up〉 configurations, respectively. The calculation results show that the vertical translation of the central ion (2.04 Å *vs.* 4.02 Å) goes along with the molecular planar framework affected as lobe-surface distances changing (3.63 Å *vs.* 2.54 Å). As a consequence, the interactions between the |Sn-dw〉 molecules and the substrate would be significantly different from those for the |Sn-up〉 molecules. DFT calculations further indicate that the total energy of the |Sn-up〉 molecules is lower than that of the |Sn-dw〉 molecules by about 1.20 eV per cell. Such an energy difference determines the adsorption energy *U*(*d*) to be an asymmetric double-well potential, which results in different conversion behaviors of the two kinds of rotors. This point that the vertical translation of the central ion goes along with the lobe-surface distances changing is different from the situation of molecules adsorbed on the metal surface where the changes of the lobe-surface distance can be negligible.^33^

In order to obtain the rotational barrier for SnPc molecules, we calculated the total energy differences between the ground state of SnPc molecules adsorbed at fcc sites and the configurations that rotate along the perpendicular rotation axis. Calculated rotational energy profiles of |Sn-dw〉 and |Sn-up〉 rotors are shown in Fig. 4(c). Here the twist angle *θ* corresponds to the angles between one of the in-plane symmetry axes of the SnPc molecules and the 〈001〉 direction of the Pt(111) surface. The calculated rotational barriers for |Sn-up〉 and |Sn-dw〉 configurations are around 187 meV and 151 meV, respectively. Once adsorption occurs, the interaction (friction) between the molecules and substrate plays a critical role in determining the molecular rotation characteristics. At a closer distance, the lobes of |Sn-up〉 molecules may simultaneously bond with the FeO surface by formation of carbon–oxygen (C–H⋯O) interaction, which leads to a high rotational energy barrier. In contrast, the lobes of |Sn-dw〉 molecules have a weaker interaction with the FeO surface due to a longer lobe-surface distance and thus show the lower rotational barrier. Accordingly, we can unequivocally assert that |Sn-up〉 is non-degenerate with |Sn-dw〉 as the obvious energy difference and Sn-up (Sn-down) molecules correspond to LR (HR) rotors. As STM does not image the atomic structure, but can resolve molecular orbitals,^44^ the closer distance between the substrate and the Sn ion in |Sn-dw〉 molecules means stronger electron hybridization (higher brightness in the STM image, as seen in Fig. 1(d)). Similar morphological characteristics also appear in the non-planar TiOPc/FeO system.^35^ During nanomanipulation, the external electric field induced by the STM tip could pump the dipolar molecules into unstable excited states in the asymmetric double-well potential, as shown in Fig. 4(d). The excited molecules have two kinds of transition paths with different probabilities. Due to the larger energy difference, it is easy for converting into the |Sn-up〉 (LR), but still has a probability of converting into |Sn-dw〉 (HR). For the free-standing SnPc molecules, the two rotors are completely degenerate in energy and possess a symmetrical double-well transition barrier with the same transition probabilities from the excited states. In our system, however, adsorption gives rise to an asymmetrical double-well transition barrier, which causes the transition of the excited molecules into two isomers with different probabilities.

**Fig. 5:**
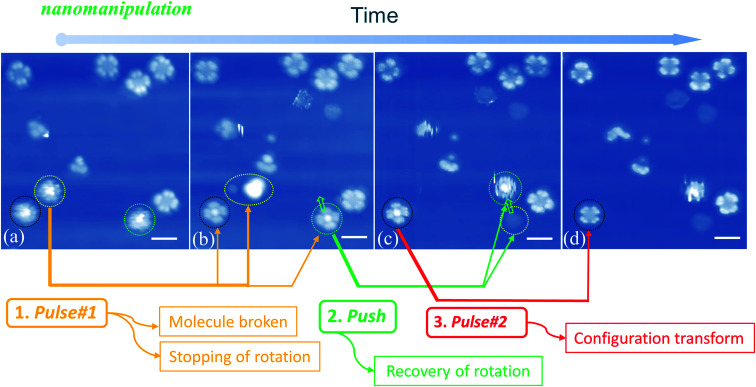
(a)–(d) STM images recorded after pulse or manipulation is applied on indicated SnPc molecules by STM tips. Step 1: applying pulse 1 breaks down the molecule marked by the yellow circle, and simultaneously two nearby molecules marked with black and green circles stop their rotational motion and exhibit four-lobe shaped features; the distances between the pulse 1 site and the two nearby molecules are about 2.6 nm and 7.8 nm, respectively. Step 2: STM tip nanomanipulation laterally pushes one of the braked molecules (green circled in (b)) to the adjacent fcc adsorption site. The molecule resumes its HR rotation behavior (green circled in (c)) and a fragment remains at the initial adsorption site (yellow circled in (c)); step 3: applying pulse 2 causes the molecular configuration transformation from the bright center to the dark center (see the molecule marked with black circle in (c) and (d)). The lines and arrows indicate the molecular transformation for every operation. Scale bars: 2 nm for all the images.

When manipulating molecular configuration conversion, we find that the molecular fragment has a great influence on the molecular rotations. Fig. 5(a) and (b) show that the molecules marked with yellow circles break down into fragments by applying current pulse #1. At the same time, we notice that two nearby molecules (circled by black and green colors, respectively) stop the rotational motion and exhibit four-lobe shaped features. The braked molecules display bright molecular centers that are obviously different from the LR rotors mentioned above in Fig. 1(d). This implies that the two braked molecules do not undergo a configuration switch and still maintain the initial Sn-down configuration. In order to confirm this point, we used the STM tip to push one of the braked molecules (the one marked with a green circle in Fig. 5(b)) to the adjacent fcc adsorption site as pointed by the green arrow in Fig. 5(c). Exactly, we find that the moved molecule resumes its HR rotation behavior (green circle in Fig. 5(c)) and a molecular fragment remains at the initial adsorption site (yellow circle in Fig. 5(c)). The observed fragment should come from the molecule smashed by pulse #1 that is indicated by a yellow circle in Fig. 5(a). It can easily diffuse along the surface due to the decoupling feature of the FeO film and then is trapped at the space between the green-circled molecules and substrate in Fig. 5(b). The stoppage of molecular rotation is likely due to the significant increase of the rotational barrier resulting from the steric hindrance of the molecular fragment. Moreover, when the current pulse #2 is applied to the black circled molecule (see Fig. 5(c) and (d)), the remaining bright-center molecule transforms into the dark-center one (*i.e.* the LR rotor). This fact further clarifies that the bright-center molecules correspond to the |Sn-dw〉 configuration and do not undergo configuration transformation when the rotation stops *via* a fragment embedding. The close-to-perfect molecular manipulation straightforwardly reveals that the rotation of the HR rotor can stop by trapping a small fragment and can be recovered by separating the molecules from the fragment.

## Methods

Experiments were carried out in a low temperature ultrahigh vacuum STM system (Unisoku, Japan) with a base pressure better than 1.0 × 10^−8^ Pa. Single-crystal Pt(111) (MaTeck, Germany) was cleaned by repeated cycles of Ar^+^ sputtering and annealing until a clean surface was obtained as checked by STM. Iron atoms were evaporated from an electron beam evaporator (Tectra, Germany) equipped with an iron rod (99.995%, Alfa Aesar). FeO ultrathin films were fabricated by oxidizing iron thin films at an oxygen pressure of 1.0 × 10^−4^ Pa and the substrate temperature of about 900 K. The temperature was monitored by using an infrared pyrometer and calibrated by using a thermal couple. Thoroughly degassed SnPcs were evaporated from a silica crucible heated at about 450 K with a deposition rate of 0.05 ML min^−1^. All the STM images were acquired at liquid nitrogen temperature (∼77 K) with electrochemically etched tungsten tips and purchased Pt–Ir tips in the constant-current mode. Differential conductance (d*I*/d*V*) measurements were obtained with the help of a lock-in amplifier (Model 830, Standford Research) with an internal modulation source. The modulation of the lock-in amplifier for d*I*/d*V* was 10 mV at 777 Hz. Since in our case bias voltages were applied to samples, modulation probes the unoccupied/occupied states of the sample in positive/negative biases. Each d*I*/d*V* curve was obtained by averaging the spectra recorded on the centers of the molecules.

DFT calculations were performed by using the Vienna *Ab initio* Simulation Package (VASP).^45^ The exchange and correlation functional with a Perdew–Burke–Ernzerhof generalized gradient corrected approximation (GGA) and ultrasoft pseudopotentials describing the electron–ion interactions was used in the calculations. The wave function was described with a plane wave basis set and an energy cutoff of 400 eV was used. The van der Waals interactions have been calculated using the DFT-D2 method of Grimme.^46^ Based on the experimental observations,^34^ the (√91 × √91)*R* ± 5.2° Moiré pattern for the FeO/Pt(111) substrate was modeled at full scale by a supercell slab consisting of two Pt atomic layers (182 Pt atoms) and one FeO(111) bilayer (73 Fe atoms and 73 O atoms); a vacuum thickness of 12.0 Å was used to separate the slabs. The gamma point was considered in the *k*-point mesh for such a big supercell. The bottom layer of Pt atoms was fixed and the upper layers of the supercell (containing 1 Pt layer and FeO bilayer) were fully relaxed. All the calculations are spin-polarized. A SnPc molecule was put on the FeO/Pt(111) substrate at different adsorption sites (fcc, hcp and top sites) with the Sn atom pointing to the surface (|Sn-dw〉) and pointing to the vacuum (|Sn-up〉) to search for the most stable adsorption configuration.

## Conclusions

We assembled non-planar SnPc molecules onto a nanotemplated FeO(111) film to form two kinds of rotors with different STM morphologies, rotational speeds and stabilities. The detailed structures of molecular rotors are studied by STM and DFT calculations. It's revealed that the rotors with high and low rotation speeds correspond to the SnPc molecules with the Sn ion pointing to FeO(111) and to the vacuum, respectively. Both kinds of rotors can be transformed into each other by applying external field stimulation. DFT calculations confirm that the total energy of the |Sn-up〉 molecules is lower than that of the |Sn-dw〉 molecules. Such an energy difference determines that the adsorption energy of SnPc is an asymmetric double-well potential, and results in different conversion behaviors of the two kinds of rotors. Furthermore, we find that the molecular fragment has a great influence on the motions of the molecules. *Via* molecular manipulation, we addressed two “braking mechanisms”: one is the transformation of adsorption configurations under the external electric field stimulus that enables the molecular rotor to stop or restart its rotation; the other is the embedded molecular fragments that can stop the molecular rotation. Our approach develops a good system for manipulating molecular rotors' properties in nanophysics and has significant value for the design of controllable molecular machines.

## Author contributions

Shuangzan Lu: data curation, writing – original draft, writing – review & editing. Min Huang: software, writing – original draft, writing – review & editing. Guodong Huang: data curation, writing – original draft. Qinmin Guo: data curation, writing – original draft. Hongxing Li: data curation, software. Jinghao Deng: data curation. Chendong Zhang: resources, supervision, writing – original draft. Yinghui Yu: conceptualization, methodology, data curtion, writing – original draft, writing – review & editing.

## Conflicts of interest

There are no conflicts to declare.

## Supplementary Material

## References

[cit1] Feynman R. P. (1992). There's Plenty of Room at the Bottom (Data Storage). J. Microelectromech. Syst..

[cit2] Kottas G. S., Clarke L. I., Horinek D. (2005). *et al.*, Artificial Molecular Rotors. Chem. Rev..

[cit3] Erbas-Cakmak S., Leigh D. A., McTernan C. T., Nussbaumer A. L. (2015). Artificial molecular machines. Chem. Rev..

[cit4] The Royal Swedish Academy of Sciences, Press Release: The Nobel Prize in Chemistry 2016, They developed the world's smallest machines, 5 Oct. 2016

[cit5] Castelvecchi D. (2017). Drivers gear up for world's first nanocar race: chemists will navigate molecular wagons along a tiny golden track. Nature.

[cit6] Fletcher M., Biglarbegian M., Neethirajan S. (2013). Intelligent system design for bionanorobots in drug delivery. Cancer Nanotechnol..

[cit7] Kay E. R., Leigh D. A., Zerbetto F. (2007). Synthetic molecular motors and mechanical machines. Angew. Chem., Int. Ed..

[cit8] Mark P. (2015). The tiniest lego: a tale of nanoscale motors, rotors, switches and pumps. Nature.

[cit9] Fraser Stoddart J. (2009). The master of chemical topology. Chem. Soc. Rev..

[cit10] Whittam R., Wheeler K. P. (1970). Transport across Cell Membranes. Annu. Rev. Physiol..

[cit11] Schliwa M., Woehlke G. (2003). Molecular motors. Nature.

[cit12] Stock D., Leslie A. G. W., Walker J. E. (1999). Molecular architecture of the rotary motor in ATP synthase. Science.

[cit13] Zhao X., Norris S. J., Liu J. (2014). Molecular Architecture of the Bacterial Flagellar Motor in Cells. Biochemistry.

[cit14] Vacek J., Michl J. (2001). Molecular dynamics of a grid-mounted molecular dipolar rotor in a rotating electric field. Proc. Natl. Acad. Sci. U. S. A..

[cit15] Horinek D., Michl J. (2003). Molecular Dynamics Simulation of an Electric Field Driven Dipolar Molecular Rotor Attached to a Quartz Glass Surface. J. Am. Chem. Soc..

[cit16] Zheng X., Mulcahy M. E., Horinek D. (2004). *et al.*, Dipolar and Nonpolar Altitudinal Molecular Rotors Mounted on an Au(111) Surface. J. Am. Chem. Soc..

[cit17] Magnera T. and Michl J., Altitudinal Surface-Mounted Molecular Rotors, in Molecular Machines, ed. T. R. Kelly, Springer, Berlin, Heidelberg, 2005, pp. 63–97

[cit18] Kobr L., Zhao K., Shen Y. (2012). *et al.*, Inclusion Compound Based Approach to Arrays of Artificial Dipolar Molecular Rotors. A Surface Inclusion. J. Am. Chem. Soc..

[cit19] Gao L., Liu Q., Zhang Y. Y. (2008). *et al.*, Constructing an Array of Anchored Single-Molecule Rotors on Gold Surfaces. Phys. Rev. Lett..

[cit20] Gao L., Du S. X., Gao H.-J. (2010). Anchoring of a Single Molecular Rotor and Its Array on Metal Surfaces using Molecular Design and Self-Assembly. Int. J. Mol. Sci..

[cit21] Zhong D., Blömker T., Wedeking K. (2009). *et al.*, Surface-Mounted Molecular Rotors with Variable Functional Groups and Rotation Radii. Nano Lett..

[cit22] Baber A. E., Tierney H. L., Sykes E. C. H. (2008). A Quantitative Single-Molecule Study of Thioether Molecular Rotors. ACS Nano.

[cit23] Tierney H. L., Murphy C. J., Jewell A. D. (2011). *et al.*, Experimental demonstration of a single-molecule electric motor. Nat. Nanotechnol..

[cit24] Tierney H. L., Murphy C. J., Sykes E. C. H. (2011). Regular Scanning Tunneling Microscope Tips Can Be Intrinsically Chiral. Phys. Rev. Lett..

[cit25] Kudernac T., Ruangsupapichat N., Parschau M. (2011). *et al.*, Electrically driven directional motion of a four-wheeled molecule on a metal surface. Nature.

[cit26] Štacko P., Kistemaker J. C. M., van Leeuwen T., Chang M.-C., Otten E., Feringa B. L. (2017). Locked synchronous rotor motion in a molecular motor. Science.

[cit27] Peller D., Kastner L. Z., Buchner T., Roelcke C., Albrecht F., Moll N., Huber R., Repp J. (2020). Sub-cycle atomic-scale forces coherently control a single-molecule switch. Nature.

[cit28] Asato R., Rapenne G. (2021). *et al.*, Molecular Rotor Functionalized with a Photoresponsive Brake. Inorg. Chem..

[cit29] Wu T. (2020). *et al.*, Tuning rotation axes of single molecular rotors by a combination of single-atom manipulation and single-molecule chemistry. Chem. Commun..

[cit30] Zhao R. (2018). *et al.*, Interlocking Mechanism between Molecular Gears Attached to Surfaces. ACS Nano.

[cit31] Abid S. (2021). *et al.*, Desymmetrised pentaporphyrinic gears mounted on metallo-organic anchors. Chem. Sci..

[cit32] Baran J. D. (2010). *et al.*, Inversion of the shuttlecock shaped metal phthalocyanines MPc (M = Ge, Sn, Pb) – a density functional study. Phys. Chem. Chem. Phys..

[cit33] Wang Y. F., Kroger J., Berndt R., Hofer W. A. (2009). Pushing and Pulling a Sn Ion through an Adsorbed Phthalocyanine Molecule. J. Am. Chem. Soc..

[cit34] Rienks E. D. L., Nilius N., Rust H.-P., Freund H.-J. (2005). Surface potential of a polar oxide film: FeO on Pt(111). Phys. Rev. B: Condens. Matter Mater. Phys..

[cit35] Lu S., Huang M., Qin Z., Yu Y., Guo Q., Cao G. (2018). Highly ordered molecular rotor matrix on a nanopatterned template: titanyl phthalocyanine molecules on FeO/Pt(111). Nanotechnology.

[cit36] Hla S. W. (2014). Tuning in to the Smallest (Man-Made) Mechanical Resonator. Physics.

[cit37] Qinmin G., Huang M., Qin Z., Cao G. (2012). Molecular orbital imaging of cobalt phthalocyanine on native oxidized copper layers using STM. Ultramicroscopy.

[cit38] Wahl M., Stohr M., Spillmann H., Jung T. A., Gade L. H. (2007). Rotation-libration in a hierarchic supramolecular rotor-stator system: Arrhenius activation and retardation by local interaction. Chem. Commun..

[cit39] Wintjes N., Bonifazi D., Cheng F., Kiebele A., Stöhr M., Jung T., Spillmann H., Diederich F. (2007). A Supramolecular Multiposition Rotary Device. Angew. Chem., Int. Ed..

[cit40] Kühne D., Klappenberger F., Krenner W., Klyatskaya S., Ruben M., Barth J. V. (2010). Rotational and constitutional dynamics of caged supramolecules. Proc. Natl. Acad. Sci. U. S. A..

[cit41] Walzer K., Hietschold M. (2001). STM and STS investigation of ultrathin tin phthalocyanine layers adsorbed on HOPG(0001) and Au(111). Surf. Sci..

[cit42] de Boer B., Hadipour A., Mandoc M. M., van Woudenbergh T., Blom P. W. M. (2005). Tuning of Metal Work Functions with Self-Assembled Monolayers. Adv. Mater..

[cit43] Lu S., Qin Z., Guo Q., Cao G. (2017). Work function mediated by deposition of ultrathin polar FeO on Pt(111). Appl. Surf. Sci..

[cit44] Gross L. (2011). Recent advances in submolecular resolution with scanning probe microscopy. Nat. Chem..

[cit45] Kresse G., Furthmüller J. (1996). Efficient iterative schemes for ab initio total-energy calculations using a plane-wave basis set. Phys. Rev. B: Condens. Matter Mater. Phys..

[cit46] Grimme S. (2006). Semiempirical GGA-type density functional constructed with a long-range dispersion correction. J. Comput. Chem..

